# βIII-Tubulin Gene Regulation in Health and Disease

**DOI:** 10.3389/fcell.2022.851542

**Published:** 2022-04-28

**Authors:** Alastair M. P. Duly, Felicity C. L. Kao, Wee Siang Teo, Maria Kavallaris

**Affiliations:** ^1^ Children’s Cancer Institute, Lowy Cancer Research Center, UNSW Sydney, Randwick, NSW, Australia; ^2^ Australian Center for NanoMedicine, UNSW Sydney, Sydney, NSW, Australia; ^3^ School of Women and Children’s Health, Faculty of Medicine and Health, UNSW Sydney, Sydney, NSW, Australia; ^4^ UNSW RNA Institute, UNSW Sydney, Sydney, NSW, Australia

**Keywords:** *TUBB3*, βIII-tubulin, microtubule, gene regulation, cancer, neuronal tubulin, tubulin, human

## Abstract

Microtubule proteins form a dynamic component of the cytoskeleton, and play key roles in cellular processes, such as vesicular transport, cell motility and mitosis. Expression of microtubule proteins are often dysregulated in cancer. In particular, the microtubule protein βIII-tubulin, encoded by the *TUBB3* gene, is aberrantly expressed in a range of epithelial tumours and is associated with drug resistance and aggressive disease. In normal cells, *TUBB3* expression is tightly restricted, and is found almost exclusively in neuronal and testicular tissues. Understanding the mechanisms that control *TUBB3* expression, both in cancer, mature and developing tissues will help to unravel the basic biology of the protein, its role in cancer, and may ultimately lead to the development of new therapeutic approaches to target this protein. This review is devoted to the transcriptional and posttranscriptional regulation of *TUBB3* in normal and cancerous tissue.

## 1 Introduction

Microtubules are one of the major constituents of the cell cytoskeleton and are made up of α- and β-tubulin heterodimers. Microtubules are highly dynamic filament structures that play critical roles in cellular processes, including vesicular transport, cell motility and mitosis. The α/β-tubulin heterodimers are made up of combinations of the different α- and β-tubulin isotypes (reviewed in Nogales 2000), of which in humans there are currently eight and seven different α- and β-tubulin isotypes, respectively (reviewed in Ludueña 2013). Each of the isotypes are encoded by a different gene and display different tissue and developmental stage expression (reviewed in Ludueña 1993; Verdier-Pinard et al., 2009). While each isotype shares high degrees of structural homology with each other, they have some differences at the peptide sequence level, specifically at their carboxy-terminal tails (Sullivan and Cleveland 1986). Sequence variations within the carboxy-terminal tails of various tubulin isotypes, have been demonstrated to regulate the dynamic assembly and disassembly of microtubule structures (Parker et al., 2018).

A strong interest exists for studying microtubules due to their importance as a target for anticancer therapies. Drugs targeting microtubules and microtubule dynamics are widely used in many cancer therapeutic regimens (reviewed in Jordan and Wilson 2004; La Regina et al., 2019). Clinically relevant Tubulin-Binding Agents (TBAs) such as the taxanes, vinca alkaloids, epothilones, and Eribulin, all bind to the β-tubulin subunits of the αβ-heterodimers (reviewed in Jordan and Wilson 2004; La Regina et al., 2019). These agents disrupt normal mitotic spindle function, block the metaphase to anaphase transition of the cell cycle, and induce mitotic arrest and cell death (reviewed in Jordan and Wilson 2004; La Regina et al., 2019). Despite the clinical success of TBAs and advances in chemotherapies, the persistent emergence of drug resistance largely hinders their clinical utility and is the primary cause of treatment failure for many cancers. Mechanisms mediating TBA resistance can occur at multiple levels (reviewed in Kavallaris, 2010; Katsetos and Draber, 2012; Parker et al., 2017). Previous studies have reported that altered expression of specific β-tubulin isotypes is strongly associated with resistance to TBAs (Kavallaris et al., 1997; Ranganathan et al., 1998a; Kavallaris et al., 1999). Of note, one particular isotype, βIII-tubulin, encoded by the *TUBB3* gene, has demonstrated aberrant expression in the clinical setting, and has been identified as a marker of drug resistance and tumour aggressiveness in a sub-set of epithelial cancers (reviewed in Kavallaris, 2010; Karki et al., 2013; Mariani et al., 2015). In addition, there is clinical evidence in lung, ovarian, glioblastoma, and breast cancer, that patients with aberrant βIII-tubulin expression exhibit poorly differentiated tumour tissue, high grade malignancy, shorter disease progression, unfavourable prognosis and worse overall survival (reviewed in Seve and Dumontet 2008; Kavallaris 2010; Katsetos et al., 2011; Katsetos et al., 2015; Mariani et al., 2015; Kanakkanthara and Miller 2021). Post-translational modifications to tubulin proteins, including βIII-tubulin, are found in normal tissue and cancer cells, and have been well described elsewhere (Ludueña 1998; Wattanathamsan and Pongrakhananon 2021; Bär et al., 2022).

Interest in βIII-tubulin is not limited to its expression in cancer. Expression of βIII-tubulin is also observed in the early stages of neurogenesis of fetal development (Caccamo et al., 1989; Lee et al., 1990b; Jiang and Oblinger 1992; Linhartová et al., 1992; Easter et al., 1993; Hausrat et al., 2021). βIII-tubulin itself is primarily thought of as a neuronal protein, observed in neurons and involved with neurogenesis and axonal growth (Caccamo et al., 1989; Jiang and Oblinger 1992; Easter et al., 1993; Tischfield et al., 2010; Latremoliere et al., 2018; Hausrat et al., 2021). This notion has been strengthened by the identification of mutations in *TUBB3*, the gene that encodes for βIII-tubulin, resulting in nervous system disorders such as Congenital Fibrosis of the Extraocular Muscles type 3 (CFEOM3), which combines the weakening of the extraocular muscles with intellectual disability, as well as axonal abnormalities and disorganisation of cortical neurons (Poirier et al., 2010; Tischfield et al., 2010). Evidence also suggests βIII-tubulin has roles outside of neurogenesis, such as the formation of neural crest cell formation (Haendel et al., 1996; Chacon and Rogers 2019) and recently, in the mineralisation stages of tooth development (Oshima and Yawaka 2020). As such, despite the original neuronal findings, βIII-tubulin expression is increasingly observed outside of neuronal tissue, with reports of adult stem cells expressing βIII-tubulin, such as melanocytes (Locher et al., 2013) and spermatogenic cells (Person et al., 2017). Additionally, the expression of βIII-tubulin in induced pluripotent stem cells has also been observed (Daily et al., 2017; Kuang et al., 2019). However, the role of βIII-tubulin in these cells remains unclear.

Despite the relevance of βIII-tubulin protein in development and cancer, there is limited information on the precise elements that regulate the gene expression of *TUBB3* in normal and cancerous human cells. This review will focus on the normal regulation of *TUBB3* transcription, the role of this gene in neurogenesis and development, and on factors contributing to the dysregulation of *TUBB3* expression in cancer and drivers of its aberrant expression. The review will present what is known and critically discuss gene regulatory elements including the drivers, or repressors, of *TUBB3* gene expression.

### 2 The Human *TUBB3* Gene Loci

Originally referred to as class III isotype β4, human βIII-tubulin was first identified in 1986 after being previously discovered in chickens a few years beforehand (Lopata et al., 1983; Sullivan and Cleveland 1986). At the time, the protein sequence of βIII-tubulin was found to be conserved across mammals, however, it was observed to be highly divergent from other β-tubulin isotypes in its carboxyl terminal region (Sullivan and Cleveland 1986). The sequence of the human βIII-tubulin gene *TUBB3* was not identified until much later, with the sequence of the more common mRNA variant being determined in 1998 (Ranganathan et al., 1998b), and its genomic location confirmed in 2010 (Tischfield et al., 2010). The *TUBB3* loci is present within the telomeric region of the long arm of chromosome 16 (Katsetos et al., 2002). Furthermore, up until 2010, *TUBB3* was also referred to as CFEOM3 when Doherty et al. (1999) first described the gene in Extraocular Congenital Fibrosis Syndrome and identified the chromosomal location of the gene through linkage analysis of DNA microsatellite markers. It was Tischfield et al. (2010) who then identified that CFEOM3 and *TUBB3* were one and the same after mapping eight different CFEOM3 causing mutations to *TUBB3*. *TUBB3* mutations are also seen and have been reported in tumours, however the impact of these mutations are unknown (reviewed in Kanakkanthara and Miller 2021).

**FIGURE 1:**
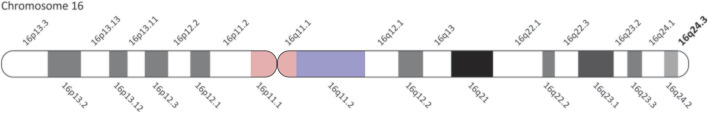
Location of the human *TUBB3* loci. Cytogenetic map of chromosome 16 showing all regions. *TUBB3* is located within 16q24.3, located at the bottom of chromosome 16 (bolded).

The human *TUBB3* gene (NG_027810.1) is 21,089 bp in length and in a genomic context, the cytogenetic location of the gene is 16q24.3 on the plus strand (Figure 1). Within this locus, the Ensembl database reports that there are 15 unique *TUBB3* transcripts (Table 1), however only 2, referred to as variants 1 and 2, have been studied. This could be due to these two having a higher abundance of mRNA than the other transcripts, with several transcripts being predicted to undergo nonsense mediated decay (Cunningham et al., 2018), or the other transcripts exist as mere sequencing artifacts. Given that *TUBB3* is primarily expressed in neuronal tissue, it is possible that these alternative transcripts of *TUBB3* represent further specialised neuronal forms of the transcript, as neuronal tissue is known to have an expanded repertoire of gene expression and alternative splicing (The GTEx Consortium, 2015; Melé et al., 2015). *TUBB3* also has two pseudogenes, *TUBB3P1* and *TUBB3P2*. *TUBB3P1* the larger of the two is located on chromosome 6, while *TUBB3P2* is located on chromosome 7. It is unknown if these pseudogenes possess any functional capacity.

**TABLE 1:** All identified human *TUBB3* transcripts.

Transcript ID	Size (bp)	Biotype	Variant (NCBI)	RefSeq	Protein	UniProt
ENST00000315491.12	1,706	Protein coding	1	NM_006,086	450aa	Q13509
ENST00000553656.5	550	Nonsense mediated decay			51aa	G3V4U2
ENST00000553967.1	736	Protein coding			164aa	G3V2N6
ENST00000554116.5	542	Processed transcript			No protein	—
ENST00000554336.5	903	Protein coding			118aa	G3V2R8
ENST00000554444.5	1,978	Protein coding	2	NM_001,197,181	378aa	Q13509
ENST00000554927.1	561	Retained intron			No protein	—
ENST00000555576.5	572	Protein coding			97aa	G3V5W4
ENST00000555609.5	1,855	Nonsense mediated decay			55aa	G3V3J6
ENST00000555810.5	767	Protein coding			189aa	G3V2A3
ENST00000556536.5	925	Nonsense mediated decay			148aa	G3V3R4
ENST00000556565.5	566	Protein coding			46aa	G3V542
ENST00000557262.5	888	Nonsense mediated decay			51aa	G3V4U2
ENST00000557490.5	806	Nonsense mediated decay			87aa	G3V3W7
ENST00000625617.2	570	Protein coding			148aa	G3V3R4

Information sourced from Ensembl database (Cunningham et al., 2018).

**FIGURE 2:**
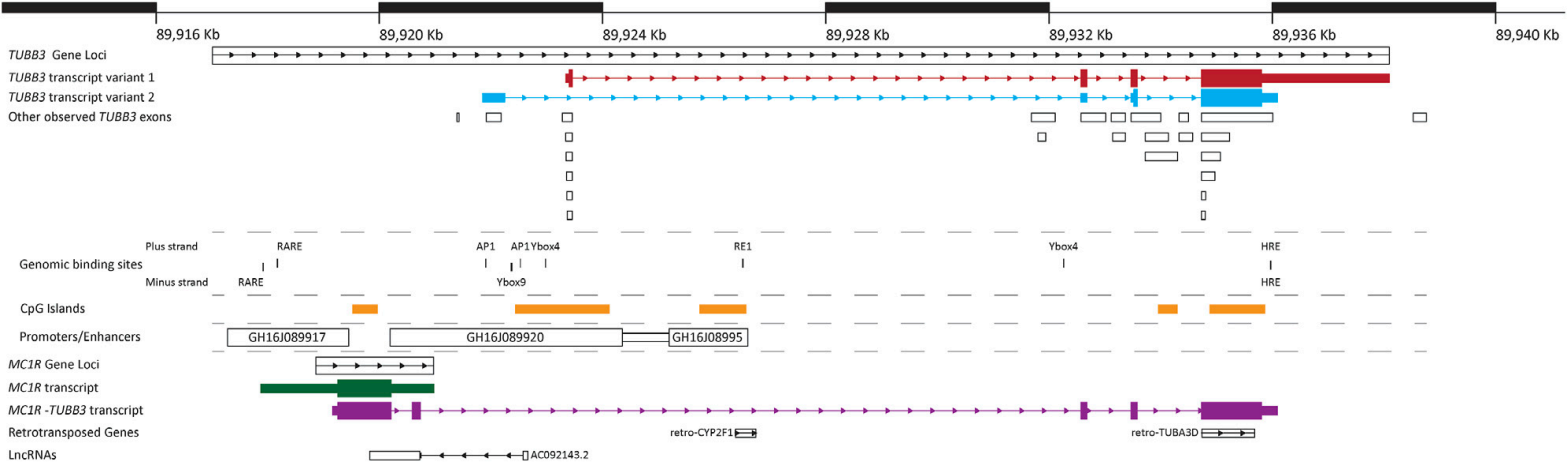
Structure of the human *TUBB3* loci. A map of the human *TUBB3* loci showing the main two transcripts of *TUBB3* as well as several other genomic structures and DNA binding sites in the region. Gene loci’s (*TUBB3*, *MC1R*, retro-CYP2F1 and retro-TUBA3D) are represented by boxes with internal arrows showing sequence direction. Individual RNA transcripts are represented by a combination of thin and thick boxes for exons, and arrows for introns; thin boxes represent untranslated regions (5′ and 3′ UTRs), while thick boxes represent translated regions. *TUBB3* transcript variants 1 and 2 are show in red and blue respectively, with addition observed *TUBB3* exons presented in white boxes; additional information of *TUBB3* transcripts is shown in Table 1. *MC1R* transcript is shown in green, the *MC1R*-*TUBB3* chimera transcript is shown in purple, and the lncRNA AC092143.2 is shown in white. Known genomic binding sites are represented with black lines, with thickness corresponding to size; additional information on known genomic binding sites is shown in Table 2 **.** CpG islands presented within the *TUBB3* loci are represented by orange rectangles. Promoters/Enhancers are shown as white boxes with their name. Locations of *TUBB3*, *MC1R* and *MC1R*-*TUBB3* transcripts, and CpG islands was extracted from the Ensembl database (Cunningham et al., 2018). Promoters/Enhancers sourced from Genehancer (Fishilevich et al., 2017). Location of retro-transposed genes and lncRNAs sourced from the UCSC genome browser (Haeussler et al., 2019). Positions based on human genome GCRh38/hg38 assembly.

From the transcripts identified for *TUBB3*, there are two main points of commonality between all of them. Firstly, the majority of the identified *TUBB3* transcripts consist of four exons, and secondly, there are two exons that are present in the majority of transcripts (Table 1; Figure 2). In transcript variants 1 and 2, these two common exons are exons 2 and 3. These two particular exons also appear in the unusual read-through product of the upstream gene encoding for the G-coupled protein receptor melanocortin 1 receptor (MC1R), which results in the formation of an unusual chimeric MC1R protein featuring the βIII-tubulin carboxyl terminus that appears to attenuate MC1R signaling (Table 1; Figure 2) (Dalziel et al., 2011; Herraiz et al., 2015) (reviewed in Herraiz et al., 2017). Further investigation into the *TUBB3* locus using alternative sequencing techniques may need to be performed to further validate these *TUBB3* transcripts, and to better understand the prevalence of this *MC1R*-*TUBB3* chimera. Because of this, only the two validated transcripts of *TUBB3* will be referred to herein.

Within the *TUBB3* loci, there are several elements. The main *TUBB3* promoter is the largest promoter found within the loci (consisting of the promoter GH16J089920 and the promoter/enhancer GH16J08995) (Fishilevich et al., 2017). Several CpG islands or regions of DNA methylation, are also observed within the loci. However, only one, which is located within the first intron of *TUBB3*, has been linked to modulating the expression of *TUBB3* (Izutsu et al., 2008; Akasaka et al., 2009; Gao et al., 2012). Binding sites for several DNA binding proteins have also been identified in the human *TUBB3* loci, the majority of which are observed on the plus strand. These include Retinoic Acid Response Elements, binding sites for AP1, a Ybox9 element and two Ybox4 elements, an RE1 site and overlapping Hypoxia Response Element (HRE) located in the 3′ region of the loci present on both the plus and minus strands (Figure 2; Table 2) (Saussede-Aim et al., 2009b; Shibazaki et al., 2012; Bordji et al., 2014; Raspaglio et al., 2014; Namekawa et al., 2020). The HRE element on the plus strand is also a canonical E-box motif, though it remains to be determined if other E-box binding proteins can bind to it. Studies performed in other animals also suggest that within the *TUBB3* loci there is an Androgen Receptor Element (ARE), an additional E-box motif, and other elements listed in Table 3 (Dennis et al., 2002; De Gendt et al., 2011). The gene *MC1R*, whose loci is observed to be wholly within the *TUBB3* loci, has a transcription region 2.5 kb upstream of the *TUBB3* transcription start site (Figure 2). The *MC1R* promotor, GH16J089917, is also found within the 5’ region of the *TUBB3* loci (Fishilevich et al., 2017).

**TABLE 2:** Validated genomic binding sites within the human *TUBB3* loci.

Element type	Genomic Location (chr 16)	Role	Identified observed in	References
Binding sites				
RARE 1^a^	89,917,926–89,917,910	Promotes expression	Cancer stem-like cells derived from patient Bladder cancer cells	Namekawa et al. (2020)
RARE 2	89,918,166–89,918,182			
AP1 binding Site 1	89,921,904–89,921,910	Promotes expression	MCF-7 breast carcinoma cells	Saussede-Aim et al. (2009b)
AP1 binding Site 2	89,921,924–89,921,930			
Ybox-9 element^a^	89,922,380–89,922,356	Promotes expression	A2780, OVCAR-3, SKOV6 and Ov2774 ovarian carcinoma cells	Raspaglio et al. (2014)
YBOX-4 element 1	89,922,975–89,922,982	Promotes expression	H522 non-small cell lung cancer cells	Castillo et al. (2012)
YBOX-4 element 2	89,932,859–89,932,866			
Rest1 binding site	89,926,504–89,926,524	Represses expression	HEK293 embryonic kidney cells; HeLa cervical cancer cells	Shibazaki et al. (2012)
Hypoxia response element^b^	89,935,970–89,935,974	Promotes expression	A2780 ovarian carcinoma cells; GL15 and U87 glioblastoma cells	(Raspaglio et al., 2008; Bordji et al., 2014)
CPG islands				
38 CPGS	89,919,506–89,919,948	Role unknown		
132 CPGS	89,922,423–89,924,099	Role unclear	OVCAR-3, JHOC-5 and JHOC-8 ovarian carcinoma cells;	Izutsu et al. (2008)
86 CPGS	89,925,728–89,926,552	Potential repressor	OVCAR-3, JHOC-5 and JHOC-8 ovarian carcinoma cells; HMV-I, HMV-II, MM-RU, SK-MEL-28, PM-WK, CRL1579, and G361 melanoma cells; NHEM-M and NHEM-D primary neonatal epidermal melanocytes	(Izutsu et al., 2008; Akasaka et al., 2009)
30 CPGS	89,933,944–89,934,279	Role unknown		
80 CPGS	89,934,864–89,935,848	Role unknown		
Promoter regions				
GH16J089917	89,917,279–89,919,454	*MC1R* and *MC1R*-*TUBB3* promoter		Fishilevich et al. (2017)
GH16J089920/GH16J08995	89,920,191–89,924,356 & 89,925,193–89,926,602	*TUBB3* promoter		Fishilevich et al. (2017)

a Elements are on the minus strand.

b HRE, is present on both the plus and minus strand.

**TABLE 3:** Transcription factors and Genomic elements associated with *Tubb3* expression in Mice and Rats.

Element type	Species Identified in	Role	Binding site validated	References
Transcription factors				
Sp1	Rat	Promotes expression	*in silico* only	(Dennis et al., 2002; Sleiman et al., 2011)
Ap2	Rat	Unknown	*in silico* only	Dennis et al. (2002)
Pea3^a^	Rat	Unknown	*in silico* only	Dennis et al. (2002)
Pit1^a^	Rat	Unknown	*in silico* only	Dennis et al. (2002)
C/EBP family	Rat	Unknown	*in silico* only	Dennis et al. (2002)
Rest1	Rat & Mouse	Inhibits expression	*in silico* only	(Dennis et al., 2002; Shibazaki et al., 2012)
Scrt1	Mouse	Promotes expression	No, potentially E-Box motifs	Nakakura et al. (2001b)
Math2	Mouse	Promotes expression	No, potentially E-Box motifs	Uittenbogaard and Chiaramello (2002)
pRB	Mouse	Promotes expression	No, potentially E-Box motifs	Toma et al. (2000)
Id2	Mouse	Inhibits expression	N/A, impairs other transcription factors binding	Le Dréau et al. (2018)
Pax3	Rat & Mouse	Inhibits expression	Yes (Rat)	(Cao et al., 2017; Wei et al., 2018)
SoxC family (Sox4, Sox11, Sox12)	Mouse	Promotes expression	Yes	(Bergsland et al., 2006; Hoser et al., 2008)
Binding elements				
E-box motifs	Rat	Unknown	*in silico* only	Dennis et al. (2002)
Central nervous system enhancer motifs	Rat	Unknown	*in silico* only	Dennis et al. (2002)
Tata box	Rat	Unknown	*in silico* only	Dennis et al. (2002)
Androgen response elements	Mouse & Rat	Promotes expression	Yes (Mice)	De Gendt et al. (2011)

a Pit1 and Pea3 sites not observed in mice (Liu et al., 2007).

Outside of the above elements associated with the expression of *TUBB3*, two retro-transposed genes are located within the loci on the plus strand (Figure 2). The first retro-transposed gene is that of *CYP2F1*, located within the first intron of *TUBB3*, and the second are elements of several exons of *TUBA3D* (Baertsch et al., 2008). Finally, on the minus strand, a single lncRNA, known as AC092143.2 or lnc-CENPBD1-3:7 exists within the *TUBB3* loci. Functionally, the potential role of these retro-transposed genes and lncRNA has yet to be investigated. In summary, the *TUBB3* loci is complex, and contains many unexplored elements that may be involved with influencing its expression, and several elements associated with *TUBB3* expression need to be mapped.

### 3 Regulation and Expression of the *TUBB3* Gene

Traditionally, βIII-tubulin has been considered a neuronal specific protein, and has been primarily used as a marker for neurons (Caccamo et al., 1989). With the advancement of sequencing approaches, it has become evident that the gene encoding for βIII-tubulin, *TUBB3,* is expressed in a wide range of tissues across the body. *TUBB3* expression is enriched in both the central and peripheral nervous systems, however, expression is also high in the testis (The GTEx Consortium, 2015). Recently, a large scale immuno-histological study was performed by Person et al. (2017) to gain a better understanding of βIII-tubulin expression across the human body in normal and cancerous tissues. Much like what was observed from sequencing studies, their work identified βIII-tubulin expression at varying amounts in the majority of human tissues, however, no comparison between expression in cancerous and normal tissues was performed (Person et al., 2017). Within the individual normal tissues, βIII-tubulin expression appeared predominantly in neurons, endothelial cells, fibroblasts and localized stem-like cells (Person et al., 2017).

As βIII-tubulin/*TUBB3* displays differential expression across different cell types across the human body (The GTEx Consortium, 2015; Person et al., 2017), it suggests that there may be unique or tissue-specific transcriptional regulatory mechanisms for *TUBB3* in different tissues. And indeed, several different mechanisms have been identified suggesting a complex nature to the regulation of *TUBB3* in normal tissue. The presence of multiple different regulatory mechanisms does suggest however, that there are multiple routes that can lead to perturbed *TUBB3* expression such as that observed in cancerous tissue. This section will focus on these mechanisms, by discussing what has been learnt about the normal regulation of *TUBB3* in healthy tissues, after which the focus will shift to what has been uncovered from studies into dysregulated *TUBB3* expression in cancer. Transcription factors with a mechanical link to the regulation of *TUBB3* have been summarised in Table 4.

**TABLE 4:** Transcription factors with known impact on *TUBB3* expression.

Transcription factor	Species	Role	References
Androgen receptor	Mouse, Human	Promotes	(Denolet et al., 2006; De Gendt et al., 2011) Butler et al. (2001)
Estrogen receptor	Human	Promotes	Saussede-Aim et al. (2009a)
HIF1α	Human	Promotes Inhibits	Raspaglio et al. (2014), Bordji et al. (2014)
HIF2a	Human	Promotes	Raspaglio et al. (2008)
ID2	Human, Mouse	Inhibits	(Le Dréau et al., 2018), Azzarelli et al. (2022)
Math2	Mouse	Promotes	Uittenbogaard and Chiaramello (2002)
MZF1	Human	Promotes	Kanojia et al. (2020)
Pax3	Rat, Mouse	Inhibits	Cao et al. (2017), Wei et al. (2018)
RARa	Human	Promotes	Namekawa et al. (2020)
RE1	Human	Inhibits	Shibazaki et al. (2012)
Scrt1	Mouse	Promotes	Nakakura et al. (2001b)
SOXC family (4, 11, 12)	Mouse, Human^a^	Promotes	(Bergsland et al., 2006; Hoser et al., 2008) (Castillo et al., 2012; Fu et al., 2019)
SOX9	Human	Promotes	Raspaglio et al. (2014)
ZEB1	Human	Promotes^b^	(Lobert et al., 2013; Kanojia et al., 2020)
ZIC1	Human	Promotes	(Fu et al. 2019)

a Only SOX4 and SOX11 have been confirmed in humans.

b ZEB1 is only implied as binding site, while identified, was not reported.

### 3.1 Drivers of *TUBB3* Expression in Normal Tissue

As mentioned, *TUBB3* displays differential expression across the body, and factors driving its expression in different cell types in normal tissue have been proposed. To date, the primary focus into what drives *TUBB3* expression in normal tissue has focused on its expression in both the central and peripheral nervous systems (CNS and PNS, respectively), and recently has been expanded to the roles *TUBB3* may be playing in neural crest cell formation during development. Additionally, the field has made some headway in understanding why *TUBB3* expression is predominantly repressed outside of neuronal tissues, and what mechanisms appear to be driving the observed *TUBB3* enrichment in the testis.

#### 3.1.1 Neurogenesis and Neural Crest Cell Formation

βIII-tubulin has been considered as one of the earliest markers of neuronal differentiation of both the CNS and PNS where it is expressed either during, or prior to, terminal mitosis of the progenitor cells. This being either neuroepithelial cells for the CNS (Caccamo et al., 1989; Lee et al., 1990b; Linhartová et al., 1992; Easter et al., 1993), or neural crest cells for the PNS (Moody et al., 1989; Haendel et al., 1996). Indeed, cloning and *in silico* characterization of the 5’ flanking region of rat *Tubb3* gene has revealed its minimal promoter region and several potential neuronal regulatory motifs (Dennis et al., 2002). This included putative binding sites for transcription factors Sp1, Ap2, Pea3, Pit1, and the C/EBP family, several E-box motifs, and a CNS enhancer motif (Table 3) (Dennis et al., 2002). There are differences in the expression of *Tubb3* between the CNS and PNS. As shown in rats and mice, *Tubb3* expression peaks during periods of axonal guidance and neuronal maturation, and then declines in the CNS with maturity while in the PNS continues to maintain high expression (Jiang and Oblinger 1992; Hausrat et al., 2021). This suggests that there may be specific regulatory mechanisms even within neuronal tissues. Indeed, several transcription factors have been identified in mouse and rat models that are involved with the expression of *Tubb3* in neurogenesis in either the CNS or the PNS (Table 3).

A regulatory candidate in the CNS is Scratch1 (*Scrt1*), a Snail family zinc finger transcription factor that is specifically expressed in post-mitotic and newly differentiating neurons (Nakakura et al., 2001a). After initially identifying the co-expression of Scratch with βIII-tubulin by treating mouse P19 embryonal carcinoma cells with retinoic acid, Nakakura et al. (2001b) discovered that overexpressing Scratch1 by itself was sufficient to induce βIII-tubulin (Nakakura et al., 2001b). Two Retinoic Acid Response Elements have been recently identified within the human *TUBB3* gene, and retinoic acid alone can induce *TUBB3* expression (Namekawa et al., 2020). Another CNS transcription factor shown to stimulate βIII-tubulin expression during neuronal differentiation is the basic helix-loop-helix differentiation transcription factor Math2 (Uittenbogaard and Chiaramello 2002; Uittenbogaard and Chiaramello 2004). Although the binding sites of Scratch1 and Math2 in the *Tubb3* loci have not been elucidated, both are known to bind to E-box motifs (Nakakura et al., 2001a; Uittenbogaard et al., 2003) and are potentially binding previously predicted sites (Dennis et al., 2002).

In addition to promoting gene expression, inhibitors of βIII-tubulin expression have also been identified in neuronal tissues. One of these inhibitors is ID2, which was originally speculated to be able to represses *TUBB3* transcription (Katsetos et al., 2003). This was because elevated Id2 was shown to interfere with retinoblastoma tumour suppressor protein’s (pRb) capacity to bind to basic helix-loop-helix transcription factors, like the *Tubb3* regulator Math2 (Uittenbogaard and Chiaramello 2002), and prevented the expression of neuronal specific genes in primary murine cortical progenitor cells (Toma et al., 2000). While alterations to *Tubb3*/βIII-tubulin expression were not examined by Toma et al. (2000), Id2 has since been demonstrated to indirectly impair *Tubb3* transcription (Le Dréau et al., 2018). More recently, ID2 levels have been shown to influence neuronal differentiation of human glioblastoma stem cells, with elevated ID2 reducing the number of βIII-tubulin positive cells (Azzarelli et al., 2022). Another inhibitor of *Tubb3* transcription is Pax3, which in rat neuronal stem cells was able to bind to the *Tubb3* promoter regions and inhibit both transcription and translation (Cao et al., 2017). Subsequent work in mouse neuronal stem cells identified that during neurogenesis, Pax3 expression was reduced through elevated levels of miR-124, which resulted in increased *Tubb3* expression and the development of neuronal phenotypes (Wei et al., 2018). In the same study, low levels of βIII-tubulin were present in these neuronal stem cells, suggesting that Pax3 partially suppresses *Tubb3* expression (Wei et al., 2018).

Specific protein 1 (Sp1) is a transcription factor predicted to have many putative binding sites in the rat *Tubb3* promoter region, suggesting it may have a potential role in the regulation of *Tubb3* expression (Dennis et al., 2002). Sp1 is also predicted to have binding sites within the human *TUBB3* promoter GH16J089920 (Fishilevich et al., 2017) (Figure 2). Sp1, a protein that is considered to be ubiquitously expressed in mammalian tissue, is known to function by binding GC-rich sequences and recruiting essential machineries to TATA boxes (one of which was also identified by Dennis et al., 2002) to initiate transcription of its target genes (Naar et al., 1998). The targeted inhibition of Sp1 activity in primary rat cortical neurons has been demonstrated to reduce the expression of *Tubb3*, alongside several other genes (Sleiman et al., 2011), suggesting that Sp1 is involved in *Tubb3* transcription activation. Although Sp1 expression decreases after neuronal differentiation and is not detected in differentiated neurons (Mao et al., 2007; Mao et al., 2009). *Tubb3* expression also declines in the CNS with neuronal maturity (Jiang and Oblinger 1992; Hausrat et al., 2021). Like Pax3, Sp1, is also a target of miR-124 (Mondanizadeh et al., 2015), although a link between Sp1, miR-124 and *Tubb3* expression has not been reported.

While the factors involving *Tubb3* expression described in the preceding section are associated with neuronal differentiation of the CNS, it is uncertain whether these shared by differentiating neurons of the PNS. During fetal development, however, βIII-tubulin has been shown to be expressed by neural crest cell progenitors (Pax7 positive cells), and by pre-migratory neural crest cells (Sox9 and Slug positive cells) prior to neurogenesis in the CNS (Chacon and Rogers 2019). Neural crest cells are considered to be multipotent progenitor cells able to give rise to various cells including neurons and melanocytes, and form the majority of the PNS (Acloque et al., 2008). This identification of βIII-tubulin expression in pre-migratory neural crest cells suggests that along with being involved with neurogenesis that βIII-tubulin has a separate role involved with neural crest cell development (Acloque et al., 2008). In this context, several factors linked with βIII-tubulin have been identified. For example, Ap2 has been linked to neural crest cell development (Mitchell et al., 1991) and identified to have several binding sites in the rat *Tubb3* promoter (Dennis et al., 2002), however whether Ap2 can promote *Tubb3* expression is yet to be determined.

The SRY-related HMG-box transcription factors of the SoxC gene family, Sox4, Sox11 and Sox12, are associated with the formation of neural crest cells (Uy et al., 2015). These transcription factors, which have been primarily linked with neuronal differentiation (Bergsland et al., 2006), are known to induce *Tubb3* expression. Bergsland et al. (2006) first identified that the 5’ Untranslated Region (UTR) of the mouse *Tubb3* gene contains three binding sites for either Sox4 or Sox11. Through increasing the expression of either Sox4 or Sox11 in developing murine embryos, Bergsland et al. (2006) observed an increase of *Tubb3* and βIII-tubulin expression, and a reduction of both *Tubb3* and βIII-tubulin when Sox4 or Sox11 was silenced through the use of siRNA. Subsequently Sox12 was also demonstrated to bind to the mouse *Tubb3* promoter, and modulate βIII-tubulin as well (Hoser et al., 2008). Sox11 had the greatest impact on βIII-tubulin expression in neurogenesis (Bergsland et al., 2006; Hoser et al., 2008), and is required for binding to NeuroG1 in order to promote *Tubb3* in early-born neurons, a process that can be inhibited through Bm2 (Chen et al., 2015). Since these three genes have been linked to neural crest formation (Uy et al., 2015), it is therefore plausible to think that the expression of *Tubb3* observed by the neural crest progenitors (Chacon and Rogers 2019) could be driven by members of the SoxC family.

The animal studies discussed in the preceding section have been invaluable in deciphering the regulatory factors during neuronal development of βIII-tubulin. Equivalent studies examining *TUBB3* expression in neuronal tissues and neural crest cells have yet to be validated in human cells, although genomic mapping has identified transcription factors that interact with the human and rat/mouse promotors. The validated human transcription factors involved with promoting *TUBB3* expression in a neuronal setting are SOX11 and ZIC1 (Fu et al., 2019). While either SOX11 or ZIC1 promotes the expression of *TUBB3*/βIII-tubulin and induces a neuronal phenotype in U87 glioblastoma cells, the expression of ZIC1 greatly enhances the impact SOX11 has on *TUBB3* expression and neuronal differentiation (Fu et al., 2019). SOX4 has also been shown to regulate *TUBB3* expression cancer (Castillo et al., 2012), and this will be discussed in a subsequent section.

#### 3.1.2 Non-Neuronal Expression Repression or Cell Cycle Dependent Expression?

One potential reason the expression of *TUBB3* is limited outside of neuronal tissue is due to the REST binding site (RE1) present within the first intronic region of *TUBB3*, which is located after the first exon of the first *TUBB3* transcript variant (Figure 2; Table 2) (Shibazaki et al., 2012). An RE1 site is also present in the 5’ UTR of rat *Tubb3* (Dennis et al., 2002). REST is a global transcriptional silencer that represses neuron-specific gene expression in non-neuronal cells (reviewed in Ooi and Wood 2007). Typically, REST forms complexes with chromatin-modifying enzymes, such as HDACs, coREST, mSin3a, MeCP2, and suppresses neuronal gene expression by epigenetic mechanisms (reviewed in Ooi and Wood 2007). Given the binding partners of REST, it is unsurprising that the human REST site located within the CpG island is found within the GH16J08995 “enhancer” (Figure 2). It is possible that in normal healthy tissue, as a result of REST binding, that this CpG island displays increased methylation, limiting the transcription of *TUBB3* and potentially accounting for the reduced expression of *TUBB3* observed in non-neuronal tissue. This needs to be further investigated as both *TUBB3* and βIII-tubulin expression is observed in several non-neuronal tissues and cells including human fetal astrocytes, melanocytes, and spermatogenic cells (Dráberová et al., 2008; Leandro-García et al., 2010; Lehmann et al., 2017; Person et al., 2017).

Prior to identifying the REST binding site in *TUBB3*, Shibazaki et al. (2012) identified that *TUBB3* expression in HEK293 and HeLa cells fluctuated with the cell cycle. *TUBB3* expression increased throughout the S phase and βIII-tubulin expression peaked in the G2/M phase, where it appeared enriched around mitotic spindles (Shibazaki et al., 2012). Immunoprecipitation studies showed that REST was no longer bound to its RE1 site in *TUBB3* during the G2/M phase, but rather rebound during the G1 phase, where *TUBB3* expression was observed to decrease (Shibazaki et al., 2012). Knockdown studies also indicated that cell-cycle dependent *TUBB3* expression is required for mitosis and normal cell growth in their cells (Shibazaki et al., 2012). This was further supported by studies that found that silencing *TUBB3* expression sensitized cancer cell lines to epothilones, a TBA that causes cells to accumulate in G2M phase of the cell cycle (Gan et al., 2011; Narvi et al., 2013). This finding suggests that *TUBB3* is not as neuronally specific as traditionally thought. Future studies are needed to better understand the role of REST in *TUBB3* regulation.

#### 3.1.3 Testis and Other Non-Neuronal Tissues

An unexpected finding from a Lewis and Cowan 1988 study was the identification of βIII-tubulin expression in mouse testis (Lewis and Cowan 1988). This finding was initially dismissed, as the βIII-tubulin antibody that was used was also known to bind to βIVb-tubulin, which at the time was considered the only β-tubulin isotype to be expressed in the testis (Lewis and Cowan 1988). Lee et al., 1990a went on to validate βIII-tubulin expression in testis using a newly developed βIII-tubulin monoclonal antibody (TUJ1). Denolet et al. (2006) later identified the altered expression of *Tubb3* in mouse Sertoli cells, “nurse” cells in the testes involved with spermatogenesis, in response to the loss of the androgen receptor. This work was then followed up by De Gendt et al. (2011) who identified several Androgen Response Elements (ARE) present in both mouse and rat *Tubb3*, and suggested that *Tubb3* plays a critical role in spermatogenesis. The location of AREs in the human *TUBB3* gene have not been reported and we cannot exclude the possibility that the association with the AR is indirect. Nevertheless, βIII-tubulin is expressed in human Sertoli cells (Person et al., 2017). Furthermore, testosterone has been shown to induce *TUBB3*/βIII-tubulin expression in human cell lines (Butler et al., 2001), suggesting that these elements potentially exist in the human *TUBB3* gene. Person et al., 2017 also observed the strongest βIII-tubulin staining in testicular tissue in the spermatogenic cells, stem cells that give rise to sperm cells (Person et al., 2017), supporting the notion of *TUBB3* being involved with spermatogenesis (De Gendt et al., 2011). In contrast, treating rat primary cortical neurons with supra-physiological doses of testosterone failed to elevate *Tubb3* expression, despite the strong expression of the androgen receptor in the same cells (Zelleroth et al., 2021).

*TUBB3* and βIII-tubulin expression has also been demonstrated to be controlled by the estrogen receptor (Saussede-Aim et al., 2009a), which could account for the observed expression in ovary tissue (Person et al., 2017). Though it is currently unclear if *TUBB3* is expressed in oogonial stem cells *in vivo*, the female equivalent of spermatogenic cells, cultured murine oogonial stem cells have been shown to express βIII-tubulin (Cao et al., 2017). The location of the estrogen response element in the human *TUBB3* loci is unknown, as it is not present in either the 5’ or 3’ UTR of *TUBB3* (Saussede-Aim et al., 2009a).

#### 3.1.4 Translational Regulation of *TUBB3* in Normal Tissues

In additional to studying the transcriptional regulation of *TUBB3*, there has also been investigations into the regulatory factors involved with the translation of, and the stability of the *TUBB3* mRNA transcript. Work performed by Theodorakis and Cleveland (1992) demonstrated that increased cytosolic levels of β-tubulins results in a reduction to β-tubulin mRNA transcripts without impacting the level of α-tubulin transcripts. Their work suggested that there is an RNA binding agent that recognizes the first 13 coding nucleotides of the various β-tubulin transcripts that is involved with RNA stabilization, however as levels of β-tubulin protein increased, this unknown binding agent loses its affinity for the RNA resulting in destabilization of the mRNA (Theodorakis and Cleveland 1992). This was demonstrated by blocking the suspected binding site, which resulted in a loss in β-tubulin RNA (Theodorakis and Cleveland 1992). As this seminal work did not address the individual β-tubulin transcripts, how this relates to individual tubulin isotypes such as *TUBB3*/βIII-tubulin remains to be investigated.

In neurogenesis, translation of *TUBB3* is also regulated in a neuronal specific manner. In mouse P19 and Neuro2a cells, RNA binding protein Tristetraprolin was shown to bind to *Tubb3* and impair its translation (Dai et al., 2015). The authors identified many neuronal mRNAs to contain binding sites for Tristetraprolin (Dai et al., 2015). By initiating neuronal differentiation in these cells, they observed a reduction in Tristetraprolin levels followed by an increase in *Tubb3* translation, a result they were able to mimic through Tristetraprolin knockdown studies as well (Dai et al., 2015). Human *TUBB3* itself does contain a potential Tristetraprolin binding site, which appears to overlap with the binding site for the members of the miR-200 family, suggesting this mechanism of regulating *TUBB3* transcription is likely to be active in the human developing nervous system as well.

### 3.2 Expression of *TUBB3* in Cancer—A Loss in Regulation

Despite the well-established link between βIII-tubulin overexpression, drug resistance and poor clinical outcomes in patients, the regulation of *TUBB3* expression in cancer cells remains poorly understood. It is becoming apparent that mechanisms driving aberrant *TUBB3* expression in tumours are complex and may vary depending on cell type and gender. Indeed, the impact of aberrant *TUBB3* expression impacts drug resistance in different types of cancer, as in ovarian and non-small cell lung cancer where elevated *TUBB3* expression is associated with drug resistance (Kavallaris et al., 1997; Kavallaris et al., 1999), while increased *TUBB3* expression in breast cancer and melanoma cells has been identified as a sign of increased drug sensitivity (Akasaka et al., 2009; Wang et al., 2013). Due to this perturbation of *TUBB3* expression in cancer, several studies have investigated whether the altered expression of *TUBB3* is a response to chemotherapeutic agents or as a result of gene dysregulation.

In cancers where *TUBB3* is overexpressed, change in gene expression is often compared to the expression of total β-tubulin. For example, in neuronal tissues βIII-tubulin expression makes up approximately 25% of the β-tubulin pool, *TUBB3* however only accounts for 4% of the total *TUBB* expression, with *TUBB4* and *TUBB2A* making up at least 90% of the total *TUBB* expression (Cleveland et al., 1990; Leandro-García et al., 2010). This trend is seen in patient tumours, where *TUBB3* only makes up a low to moderate proportion of the *TUBB* mRNA pool in ovarian, breast and lung cancer (with proportions ranging up to 7.5, 18, and 16% respectively) (Leandro-García et al., 2010). What makes this change aberrant though is that between normal and cancerous tissue, this change in *TUBB3* expression accounts for a 71- and 43-fold increase in expression in lung and breast cancer respectively (Leandro-García et al., 2010).

#### 3.2.1 Impact of Chemotherapy on *TUBB3* Expression

Induction of *TUBB3* expression has been widely reported in numerous cancer cell lines by both short term (Ranganathan et al., 1998b) and long term (Ranganathan et al., 1996; Kavallaris et al., 1997; Ranganathan et al., 1998a; Ranganathan et al., 1998b; Shalli et al., 2005) exposure to TBAs, a class of chemotherapeutics that target tubulin and microtubule dynamics (reviewed in Jordan and Wilson, 2004; La Regina et al., 2019). The factors responsible for this response may not be unique to βIII-tubulin as the levels of several other β-tubulin isotypes were also significantly increased (Ranganathan et al., 1996; Ranganathan et al., 1998a; Shalli et al., 2005). These results should be interpreted with caution though as very high doses of TBAs were used in some of the short-term studies. For example, in MCF7 cells, *TUBB3* gene expression has been shown to be inducible following acute exposure to extremely high concentrations of vinorelbine, vinblastine or colchicine (1 μm), or paclitaxel (400 nm) (Saussede-Aim et al., 2009b; Lobert et al., 2011). Concentrations of vinblastine at 1 μm are known to completely depolymerise microtubules and increase microtubule polymer mass *in vitro* (Jordan et al., 1991; Toso et al., 1993). The concentration used is not clinically relevant and the Vinca alkaloid-induced *TUBB3* expression is likely to be a compensatory response to microtubule depolymerisation, or an “off- target” effect on the transcriptional machinery or signalling pathways. Using this extreme dose of vinorelbine or vinblastine (1 μm) in mutagenesis studies, Saussede-Aim et al. (2009b) reported that Vinca alkaloid treatments were enhancing *TUBB3* promoter activity *via* two AP1 binding sites located within the GH16J089920 promoter of the *TUBB3* loci (Figure 2; Table 2) (Saussede-Aim et al., 2009b). However, using chromatin immunoprecipitation (ChIP) for canonical AP1 binding transcription factors failed to identify what was binding to the AP1 site in response to vinorelbine exposure, suggesting that there was a non-canonical AP1 binding protein inducing *TUBB3* expression in response to vinorelbine (Saussede-Aim et al., 2009b). Future investigation using ChIP is required to identify transcription factors responsible for Vinca alkaloid-induced *TUBB3* expression at clinically relevant doses.

Like Vinca alkaloids, Taxol has been reported to alter the level of *TUBB3* expression in tumours. For example, Kavallaris et al. (1997) reported that, while the level of individual β-tubulin isotypes remained the same in normal ovary and primary untreated ovarian tumours, analysis of ovarian carcinoma specimens from the same patient before and after chemotherapy revealed that *TUBB3* and *TUBB2C* gene expression increased significantly in Taxol-resistant tumours post-treatment (Kavallaris et al., 1997). As patients develop Taxol resistance after several cycles of Taxol/platinum combination therapy, it is difficult to differentiate whether the increased *TUBB3* expression observed was a direct consequence of chemotherapy-induced changes, or as a result of selection of resistant cell populations where altered tubulin expression provided a survival advantage. Kavallaris et al. (1999) went on to show that Taxol resistant non-small cell lung cancer cells were overexpressing *TUBB3* and βIII-tubulin, and that partial suppression of *TUBB3* using antisense oligonucleotides sensitized cells to Taxol (Kavallaris et al., 1999), linking *TUBB3*/βIII-tubulin expression with Taxol sensitivity. Later, potent knockdown of *TUBB3* using siRNA and shRNA confirmed a direct functional role for βIII-tubulin in mediating *in vitro* and *in vivo* sensitivity to broad classes of chemotherapy in non-small cell lung cancer, identifying βIII-tubulin as a survival factor in cancer cells (Gan et al., 2007; Gan et al., 2010b; McCarroll et al., 2010; Gan et al., 2011).

#### 3.2.2 The 5’ Region of *TUBB3* in Cancer

Several regulatory elements in addition to AP1 binding sites mentioned earlier, have been identified within the 5’ UTR of the *TUBB3* loci, which includes two CpG islands. The shorter of the two (consisting of 38 CpGs) is located just upstream of the GH16J089920 promoter, while the second CpG island, and also the largest in the *TUBB3* loci (132 CpGs), is located within the promoter and covering the first exon of the first *TUBB3* transcript variant (Figure 2; Table 2). The larger CpG island has been identified as hypomethylated in several ovarian cancer cell lines, but not in non-cancerous ovarian tissues (Izutsu et al., 2008). Given the identification of multiple SP1 and AP2 binding sites within the rat genome around the first exon of *Tubb3* (Dennis et al., 2002), Izutsu et al. (2008) suggested that these sites may be present at similar locations of the human *TUBB3* loci too. Since SP1 and its DNA-binding activities are inducible under oxidative stress and DNA-damage (Ryu et al., 2003), and assuming there are SP1/AP2 binding sites within this region as suggested by Izutsu et al. (2008), it is possible that under chemotherapeutic insults, hypomethylated *TUBB3* promoter regions with enhanced SP1 signalling may contribute to aberrant *TUBB3* expression in ovarian cancer. Further studies are required to clarify whether SP1 and AP2 can directly bind to those hypomethylated regions and drive aberrant *TUBB3* expression. Moreover, it will be important to determine if hypomethylation of *TUBB3* occurs in patient samples with upregulated βIII-tubulin expression.

There are two Retinoic Acid Response Elements (RARE) upstream of the smaller CpG island, towards the extreme 5′ end of the *TUBB3* loci and within the *MC1R* promoter region (Figure 2; Table 2). RAREs are bound to by the transcription factor Retinoic Acid Receptor α (RARα) in response to elevated levels of retinoic acid, resulting in gene expression. The two RAREs within the *TUBB3* loci were recently discovered by Namekawa et al. while trying to improve the generation of long-term cultures of Patient Derived Cancer cells (PDCs) that were enriched for Cancer Stem-like Cells (CSCs) from surgically removed bladder tumours (Namekawa et al., 2020). CSCs are renewable cells that constitute a small population within a cancerous cell population, and are implicated in tumour drug resistance, as well as tumour recurrence and metastasis (reviewed in Clevers 2011; Diaz and Leon 2011). These PDCs were grown in a 3D spheroid culture to aid in CSC enrichment, and were observed to have elevated expression of *ALDH1A1*, a marker for CSCs and whose protein product RALDH1 oxidises retinaldehyde into retinoic acid (Namekawa et al., 2020). Knockdown studies of *ALDH1A1* showed that its expression was required for the *in vitro* maintenance of the PDCs, and prevented spheroid formation, leading the authors to speculate that spheroid formation was occurring due to elevated levels of retinoic acid caused by elevated *ALDH1A1* (Namekawa et al., 2020). After demonstrating that spheroid formation was reliant on retinoic acid levels independently of *ALDH1A1* expression, Namekawa et al. (2020) proceeded to search for genes that were being upregulated by the retinoic acid response pathway. By performing ChIP for RARα, they identified two RAREs in the 5’ UTR of the *TUBB3* loci, which were then confirmed to be able to promote the expression of *TUBB3*, and that *TUBB3* expression was elevated by the PDCs too (Namekawa et al., 2020). Subsequent knockdown studies of *TUBB3* in PDCs confirmed it as downstream to the elevation of *ALDH1A1* expression, as *TUBB3* expression was required for *in vitro* spheroid formation (Namekawa et al., 2020). This prompted the suggestion that *TUBB3* expression may contribute to the maintenance of CSCs in bladder cancer (Namekawa et al., 2020), which could account for why elevated *TUBB3* is observed with more aggressive subtypes of bladder cancer (Hinsch et al., 2017).

Recent work has identified that MZF1 is able to bind to the *TUBB3* loci, with three potential sites predicted up to 600 base pairs upstream of the first exon of *TUBB3* transcript variant 2 (Kanojia et al., 2020). The ability of MZF1 to bind to the *TUBB3* loci was identified while looking for means to upregulate βIII-tubulin expression in HER2 positive breast cancer in an effort to induce sensitivity to the TBA, vinorelbine (Kanojia et al., 2020). Building on previous work that identified *TUBB3* expression to be modulated by the members of the Bromdodmain and Extraterminal (BET) protein family (Piunti et al., 2017), Kanojia et al. (2020) identified increased *TUBB3*/βIII-tubulin expression in response to BET inhibition which led to increased sensitivity to Vinorelbine both *in vitro* and *in vivo* (Kanojia et al., 2020). Seeking a mechanism to account for why BET inhibition was promoting *TUBB3* expression, the *TUBB3* promoter (GH6J089920) was scanned and led to the identification of several potential binding sites for transcription factors (Kanojia et al., 2020). As MZF1 was associated with better survival in breast cancer patients, and because *MZF1* expression decreased upon treatment with BET inhibitors, Kanojia et al. (2020) performed knockdown/overexpression studies and ChIP-qPCR, confirming that MZF1 could bind to the *TUBB3* loci and repress *TUBB3*/βIII-tubulin expression.

Near the AP1 sites, exist two more transcription factor binding sites within the 5′ UTR of the first *TUBB3* transcript variant, both of which are Ybox elements (Figure 2; Table 2). Ybox elements are canonically bound to by *SRY*-related HMG-box transcription factors, and as mentioned earlier several of these transcription factors have been linked to modulating *TUBB3* expression in a neuronal setting. Two of these transcription factors have been linked to modulating *TUBB3* expression in cancer, SOX4 and SOX9 (Castillo et al., 2012; Raspaglio et al., 2014). While both are linked to neurogenesis and neuronal crest cell formation (Bergsland et al., 2006; Martini et al., 2013; Uy et al., 2015), increased SOX4 expression is also commonly linked with several forms of cancer, in particular lung cancer, and has been suggested as a driver oncogene (Liu et al., 2006; Castillo et al., 2012). Despite its increase in expression, the impact of increased SOX4 expression on the genes it was upregulating was unknown. To address this issue, Castillo et al. (2012) investigated genes that were positively regulated by SOX4 expression in small cell lung cancer through knockdown studies. After identifying several potential genes downregulated upon knockdown of *SOX4* expression, Castillo et al. (2012) screened these genes for potential SOX4 binding sites (Scharer et al., 2009), and subsequently confirmed SOX4 binding through ChIP and qPCR of the SOX4 bound sequences (Castillo et al., 2012). As Sox4 had been previously linked to regulating *Tubb3*/βIII-tubulin expression in neurogenesis (Bergsland et al., 2006), the authors used *TUBB3* as a positive control for their assays as it was downregulated in the initial *SOX4* knockdown microarray, and they went on to validate two SOX4 binding sites in the *TUBB3* loci (Figure 2; Table 2) (Castillo et al., 2012). Of note, the Ybox4 element identified in the 5’ region was determined to be the more dominant (Figure 2) (Castillo et al., 2012). Thus Castillo et al. (2012) reported that the dysregulation of a direct factor associated with neurogenesis in cancer may be involved with promoting the aberrant expression of *TUBB3* in some cancers. Due to its association with hypoxic stress response, the Ybox9 element and SOX9 will be discussed in a subsequent section.

#### 3.2.3 The First Intron of *TUBB3*—Epigenetic Dysregulation or Loss of REST1 Expression?

Epigenetic dysregulation is a common feature of cancers (Hanahan 2022). Like many genes, *TUBB3* can be epigenetically regulated. REST-mediated mechanisms and chromatin remodelling have been demonstrated to play an important role in *TUBB3* regulation in several epithelial cancer cells (Izutsu et al., 2008; Akasaka et al., 2009; Gao et al., 2012; Shibazaki et al., 2012). For example, in ovarian cancer cells, DNA demethylation CpG island (containing 86 CpGs) within *TUBB3* intron 1 has been shown to result in βIII-tubulin overexpression, with chromatin acetylation accelerating the process and increasing *TUBB3* expression as well (Izutsu et al., 2008; Akasaka et al., 2009). Subsequently, Izutsu et al. (2008) performed *in silico* analysis’ within this region and identified the RE1 site, later validated by others (Shibazaki et al., 2012), suggesting REST may also be involved with the observed increase in *TUBB3* expression. Follow-up investigations of this predicted RE1 site by Akasaka et al. (2009) identified that histone deacetylation of this RE1 motif partially contributes to *TUBB3*/βIII-tubulin overexpression in melanoma.

The loss of REST in a range of cancers has also been linked to the aberrant expression of neuronal genes in the clinic, including *TUBB3*. A negative correlation between REST and *TUBB3* expression has been reported in skin, ovarian, and small cell lung cancer biopsy samples (Akasaka et al., 2009; Kreisler et al., 2010; Hatano et al., 2011; Gao et al., 2012), while in normal non-neoplastic tissues *TUBB3* is barely detectable. Additionally, *REST* gene deletion and frame-shift mutations are frequently observed in colon and small cell lung cancers (Coulson et al., 2000; Westbrook et al., 2005). In mouse colonic crypts, targeted *Rest* genetic ablation has resulted in upregulation of *Tubb3* expression (Hatano et al., 2011; Gao et al., 2012). Furthermore, *TUBB3*/βIII-tubulin expression can be independently induced upon *REST* siRNA treatment in cancer cells (Akasaka et al., 2009; Gao et al., 2012). Together, these findings suggest REST as a transcriptional silencer of *TUBB3* and that dysfunctional REST, in conjunction with epigenetic modifications in *TUBB3* intron 1, may be important mechanisms underlying aberrant *TUBB3* expression in tumours of non-neuronal origin. Since other neuronal differentiation factors mentioned previously are also linked to this altered *TUBB3* expression (Castillo et al., 2012; Raspaglio et al., 2014; Namekawa et al., 2020), it poses the question—are dysregulated processes associated with neuronal gene regulation the primary causes of aberrant *TUBB3* expression in tumours of non-neuronal origin? Further research is required in order to better understand the role that these neuronal factors are playing in *TUBB3* expression in cancer.

#### 3.2.4 The 3’ UTR of *TUBB3*—Stress Response

From the observed increases in *TUBB3* expression in response to exposure to TBAs, (Ranganathan et al., 1996; Kavallaris et al., 1997; Ranganathan et al., 1998a; Ranganathan et al., 1998b; Shalli et al., 2005), one can speculate that the induction of βIII-tubulin could enable tumour cells to adapt and survive in a stressful microenvironment. Gan et al. (2007) provided the first evidence that expression of *TUBB3*/βIII-tubulin was a survival factor that when suppressed using gene silencing not only sensitized tumour cells to TBAs but also to broad classes of drugs including DNA-damaging agents and antimetabolites. A notion that is strengthened by the observation that the levels of *TUBB3* were able to modulate the PTEN/AKT signaling axis (McCarroll et al., 2015a), a prosurvival pathway commonly perturbed in a range of tumours (reviewed in Song et al., 2012; Taddei et al., 2012). Indeed, growing evidence suggests that βIII-tubulin expression is a key adaptive response that is activated on cellular exposure to a stressful microenvironment, such as hypoxic conditions (Raspaglio et al., 2008; Forde et al., 2010; Danza et al., 2012; Bordji et al., 2014; Raspaglio et al., 2014) or glucose deprivation in cancers cells (Parker et al., 2016). In solid tumours, cells often grow within a hypoxic microenvironment, and cells with a highly efficient hypoxia-inducing factor orchestrated survival program possess an advantage to offset its selective pressure.

In tumours, the hypoxia-inducing factor HIF1α has been implicated in the transcriptional regulation of βIII-tubulin via the 3′UTR of the *TUBB3* gene and is thought to protect tumours against hypoxic injury (Raspaglio et al., 2008; Forde et al., 2010; Danza et al., 2012; Bordji et al., 2014; Raspaglio et al., 2014). In A2780 ovarian cancer cells, hypoxia has been shown to strongly induce *TUBB3* gene and βIII-tubulin protein expression and this phenotype was directly linked to cisplatin and paclitaxel resistance (Raspaglio et al., 2008; Raspaglio et al., 2014). This process was shown to be transcriptionally regulated through the binding of HIF1α to a hypoxia response element (HRE) within the 3′ UTR of *TUBB3* (Raspaglio et al., 2008) (Figure 2; Table 2). An alternative transcriptional mechanism regulating *TUBB3*, involving HIF2α and the SoxC gene SOX9, has also been described (Raspaglio et al., 2014). In ovarian cancer specimens, high levels of *TUBB3* mRNA and βIII-tubulin protein were significantly associated with increasing levels of SOX9 and HIF2α (Raspaglio et al., 2014). Silencing both SOX9 and HIF2α abrogated this hypoxia-activated *TUBB3* expression, suggesting roles for SOX9 and HIF2α as positive *TUBB3* regulators under hypoxic conditions. Subsequent *in silico* analysis and ChIP studies demonstrated the binding of SOX9 to a specific binding site (the Ybox9 element mentioned earlier) within the 5′ region of *TUBB3* (Figure 2; Table 2), with gene-reporter and site-directed mutagenesis studies all supporting the involvement of SOX9 in *TUBB3* regulation in hypoxia (Raspaglio et al., 2014).

HIF1α and HIF2α may potentially regulate *TUBB3* expression in hypoxic conditions by mechanisms that differ in diverse cancer types. While both appear to have a positive impact on expression in ovarian cancer (Raspaglio et al., 2008; Raspaglio et al., 2014), HIF1α appears to play an inhibitory role on *TUBB3*/βIII-tubulin expression in glioblastoma cells (Bordji et al., 2014). In glioblastoma hypoxia reduced HIF1α expression, leading to HIF2α binding to the two overlapping HREs located in the 3′UTR of the gene (Bordji et al., 2014). Additionally, epigenetic regulation could account for this regulation in specific cancer cell lines, as hypomethylation of the HRE is required for *TUBB3* expression in ovarian cancer cells, prostate cancer cells and prostate tumours (Raspaglio et al., 2008; Forde et al., 2010). This suggests that both HIF1α and HIF2α/SOX9 mediated *TUBB3* regulation could be a cell-specific response, as it is not inducible upon hypoxia in some cell lines expressing high basal levels of βIII-tubulin (Raspaglio et al., 2008; Shen and Yu 2008; Danza et al., 2012; Levallet et al., 2012; Bordji et al., 2014; Raspaglio et al., 2014).

#### 3.2.5 miR-200c and HuR—Partners in Crime

**FIGURE 3:**
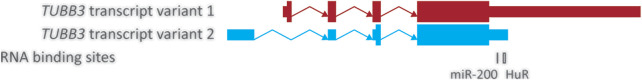
Structure of the common *TUBB3* mRNA transcripts. Structure of the two common *TUBB3* transcripts showing the validated binding sites of the HuR protein and the miR-200 family. Transcripts are represented by a combination of thin and thick boxes for exons, and arrows for introns; thin boxes represent untranslated regions (5′ and 3’ UTRs), while thick boxes represent translated regions. *TUBB3* transcript variants 1 and 2 are show in red and blue respectively and have been aligned to show common regions. RNA binding sites represented by boxes under their approximate location, with thickness corresponding to size. HuR binding site validated by (Prislei et al., 2013); miR-200c binding confirmed by (Cochrane et al., 2009); miR-200b binding confirmed by (Wu et al., 2020); miR-429 binding predicted by (Susanna et al., 2011); miR-200c binding confirmed by (Cochrane et al., 2009); miR-200b binding confirmed by (Wu et al., 2020); miR-429 binding predicted by (Susanna et al., 2011).

Another common mechanism used by cells as a means of translational regulation are microRNAs (miRNAs), small non-coding RNAs that can modulate the post-transcriptional regulation of gene expression through modulation of mRNA stability and translational efficiency through complementary base pair binding (Goodall et al., 2013; Maciotta et al., 2013). One particular family of miRNAs, the miR-200 family, has been linked to modulating the translation of *TUBB3* in the context of cancer. The miR-200 family, consisting of miR-141, −200a, −200b, −200c, and −429, have an established role in cancer, with their downregulation being linked to angiogenesis, drug resistance and the epithelial-mesenchymal transition of cancer cells (Mongroo and Rustgi 2010; Pecot et al., 2013; Brozovic et al., 2015; Sulaiman et al., 2016). The expression of all five members of this miRNA family have been shown to inversely correlate with the levels of *TUBB3* in ovarian cancer patients (Susanna et al., 2011), however only two of them, miR-200b and −200c, have been demonstrated to directly bind to *TUBB3*, while miR-429 is predicted to do so (Cochrane et al., 2009; Susanna et al., 2011; Wu et al., 2020). Given these miRNAs are from the same family, they all share a similar seed sequence and are able to bind to *TUBB3* at the same location (Figure 3).

The most well studied member of the miR-200 family in regards to *TUBB3,* is miR-200c, which has also been shown to have an interesting relationship with the RNA binding protein HuR in its modulation of *TUBB3* translation (Cochrane et al., 2009; Cochrane et al., 2010; Raspaglio et al., 2010). Cochrane et al. (2009) and Cochrane et al. (2010) found that miR-200c binds to the *TUBB3* 3’ UTR (Figure 3) which results in a reduction of βIII-tubulin without impacting the expression of *TUBB3* (Cochrane et al., 2009; Cochrane et al., 2010). In the context of cancer, identification of miR-200c regulating *TUBB3* expression came from *in vitro* work examining reduced miR-200c in model breast, ovarian and endometrial cancer cell lines (Cochrane et al., 2009; Cochrane et al., 2010). Changes in miR-200c have also been reported in a number of cancer cell lines and clinical specimens. Specifically, several separate studies reported that low miR-200c expression is significantly associated with high βIII-tubulin protein levels, resistance to TBAs, high incidence of recurrence and poor survival in ovarian cancer patients (Susanna et al., 2011; Brozovic et al., 2015; Sulaiman et al., 2016). These findings suggest miR-200c negatively regulates *TUBB3* expression and loss of miR-200c may result in βIII-tubulin overexpression in ovarian, breast and endometrial cancer. Additionally, recent work has demonstrated that intratumour delivery of miR-200c overexpressing exosomes can target *TUBB3* in *in vivo* models of tongue squamous cell carcinoma and restore tumour chemosensitivity (Cui et al., 2020), suggesting miR-200c has potential as a therapeutic strategy to treat individuals with βIII-tubulin overexpressing tumours.

In contrast, another study examined miR-200c expression in patients with ovarian cancer and found no relation between elevated miR-200c, βIII-tubulin levels, or chemotherapy sensitivity, leading them to examine additional elements involved with βIII-tubulin translation (Prislei et al., 2013). One element Prislei et al. (2013) chose to focus on was the expression of the RNA binding protein HuR, that had been associated with promoting the translation of *TUBB3* (Raspaglio et al., 2010). In ovarian cancer, Raspaglio et al. (2010) identified that while hypoglycaemic conditions caused an increase in *TUBB3* expression, the expression of βIII-tubulin in these conditions was reliant on the stabilization of the *TUBB3* transcript by cytosolic HuR binding to its 3’ UTR (Figure 3) (Raspaglio et al., 2010). The authors additionally identified that high cytosolic levels of HuR in tumours was associated with high βIII-tubulin expression and poor survival in ovarian cancer patients (Raspaglio et al., 2010). Building on this work, Prislei et al. (2013) divided up their patient cohort into those with high cytosolic or high nuclear HuR expression. They found that those with high cytosolic HuR expression with elevated miR-200c levels unexpectedly had elevated βIII-tubulin levels, exhibited chemotherapy resistance and poor patient outcomes (Prislei et al., 2013). *In vitro* work then identified that miR-200c was capable of recruiting cytosolic HuR to its binding site on the *TUBB3* transcript (Figure 3), resulting in further stabilization of the *TUBB3* transcript which potentially accounts for the higher expression of βIII-tubulin observed in patients (Prislei et al., 2013). How miR-200c impacts the recruitment of HuR to the *TUBB3* transcript is unclear and understanding this relationship would be beneficial to unravelling how *TUBB3* expression is modulated by miRNAs.

#### 3.2.6 Un-mapped Regulatory Elements of *TUBB3*

While the previous section highlighted elements that have been mapped to the *TUBB3* loci, there are several elements that have been demonstrated to regulate *TUBB3* expression but have no clear binding to the *TUBB3* loci. Mentioned earlier, the gonadal steroids estrogen and testosterone have both been shown to induce *TUBB3* expression and have emerged as potential drivers of *TUBB3*/βIII-tubulin expression in cancer (Butler et al., 2001; Saussede-Aim et al., 2009a; Mariani et al., 2012), however estrogen and androgen receptor elements (ERE and ARE respectively) in the *TUBB3* loci not been identified. In breast cancer cells, Saussede-Aim et al. (2009a) described an estrogen-dependent *TUBB3* regulatory pathway, where *TUBB3*/βIII-tubulin expression was inducible upon oestradiol exposure. While *in silico* analysis of the 5′ and 3’ UTRs of the *TUBB3* loci failed to identify where the location of any EREs were, several binding sites for transcription factors known to be implicated in indirect estrogen-regulation such as AP1, NF-κB, and SP1 were identified in the first intron of *TUBB3* (Saussede-Aim et al., 2009a). In the same study, oestradiol-induced *TUBB3* expression could not be reproduced in estrogen receptor (ER) negative breast cancer cell lines, and was abrogated after exposure to the ER antagonists tamoxifen and fulvestrant in several ER-expressing breast cancer cell lines. These findings suggest that oestradiol-induced *TUBB3* expression is ER-dependent. The authors proposed that ERs may regulate *TUBB3* in an indirect manner, facilitating transcription factor binding to nearby corresponding sites in intron 1 and subsequent *TUBB3* transcription activation. Conflicting results were reported in invasive breast cancer specimens, where high *TUBB3* expression was identified in both ER positive and ER negative breast tumour specimens (Wang et al., 2013), raising the question as to whether ER is relevant to *TUBB3* regulation in the clinic. This disparity could be explained by the different biology in cell models and clinical specimens. In the study by Wang et al. (2013), specimens were collected from patients with different pathological stages, with or without neoadjuvant chemotherapy, all of which could potentially contribute to high *TUBB3* expression. In addition, patients in this study were not treated with estrogen and therefore further studies are required to assess the clinical value of ER in *TUBB3* regulation in breast cancer.

In colorectal cancer, elevated *TUBB3* expression is associated with invasive phenotypes in both genders (Portyanko et al., 2009; Zhao et al., 2016). *In vitro* analysis of 23 colorectal cancer cell lines suggested that *TUBB3*/βIII-tubulin is activated after exposure to androgens in males (Mariani et al., 2012), as seen with estrogens in breast cancer cells (Saussede-Aim et al., 2009a). In both male and female colorectal cancer cell lines, stable silencing of androgen receptors (AR) yielded significant downregulation of *TUBB3*/βIII-tubulin, raising the possibility that ARs play a significant role in driving *TUBB3* expression. Importantly, in male colorectal cancer cells, the AR-dependent *TUBB3* regulatory pathway is constitutively activated via testicular androgen, while in colorectal cancer cell lines derived from women *TUBB3* is only inducible upon serum starvation (Mariani et al., 2012). This finding suggests that for males and females, there are differences in how the AR regulatory regions are impacted and are able to induce *TUBB3* expression in response to external stimuli. While mapped in mice and rats (De Gendt et al., 2011), future mutagenesis and ChIP studies are required to identify AR binding regions within the human *TUBB3* gene to understand this sex based expression pattern of *TUBB3*.

Other factors have also been proposed to play a role in *TUBB3* regulation. For example, overexpression of Semaphorin-6A (*SEMA6A*) is correlated with *TUBB3*/βIII-tubulin upregulation in ovarian cancer cells, while the reverse is observed in *SEMA6A* knockdown cells (Prislei et al., 2008). Likewise, levels of the transcription factor ZEB1 have also been shown to influence *TUBB3* expression in ovarian cancer in the same manner as Semaphorin-6A (Lobert et al., 2013). Additionally, Kanojia et al. (2020) identified a potential ZEB1 binding site within the *TUBB3* 5’ UTR, and their data supports that ZEB1 promotes *TUBB3* expression, as increasing *ZEB1* expression led to an elevation of *TUBB3* expression. In contrast to Semaphorin-6A and ZEB1, the overexpression of the Snail family zinc finger transcription factor SLUG in non-small cell lung cancer cells suppressed expression of *TUBB3*/βIII-tubulin, as well as the β-tubulin isotype, *TUBB4A*/βIVa-tubulin (Tamura et al., 2013). This study then focused on the relationship between Slug and *TUBB4A*, and did not investigate the SLUG induced suppression of *TUBB3* further (Tamura et al., 2013).

Slug is co-expressed with βIII-tubulin and Sox9 in pre-migratory avian neural crest cells (Chacon and Rogers 2019). Additionally, SLUG has been shown to directly interact with SOX9 to promote the formation of cancer stem-like cells in lung cancer (Luanpitpong et al., 2016), and there is the recent speculation that *TUBB3* may be playing a role in the maintenance of cancer stem like cells (Namekawa et al., 2020). Though it does present as an oddity, these studies suggest that Slug may only be a *TUBB3* repressor under certain conditions. Two other family members, Snail and Scratch1, are also involved in *TUBB3* regulation. As previously mentioned, Scratch1 expression results in increased βIII-tubulin in a neuronal setting (Nakakura et al., 2001b). In contrast, Scratch1 may not be involved with *TUBB3* regulation in a cancer setting due to its lack of expression in a wide range of patient samples obtained from different tumours (Bastid et al., 2010). The expression of the third family member, Snail/*SNAI1* itself, has also been shown to correlate with the expression of *TUBB3*/βIII-tubulin in colon cancer cells (Sobierajska et al., 2016), however other than expression, no mechanistic study has been reported. Snail and Slug both present as interesting regulators of *TUBB3*, as they are both expressed in a large range of cancers (Bastid et al., 2010), and also because of their roles in the epithelial–mesenchymal transition in tumour cells, the process linked with metastasis (Thiery and Sleeman 2006). Additional studies are required to assess whether Snail or Slug can directly bind to the *TUBB3* loci and regulate its expression, especially given βIII-tubulin’s roles in drug resistance and tumor aggressiveness.

Finally, K-Ras signalling has been associated with the regulation of βIII-tubulin translation in cancer (Levallet et al., 2012). While investigating K-Ras signalling in non-small cell lung cancer, Levallet et al. (2012) identified K-Ras mutations in clinical samples were strongly and frequently associated with positive βIII-tubulin expression. In immortalised human bronchial cells, expression of a K-Ras mutant protein was shown to significantly increase βIII-tubulin protein levels, while *TUBB3* mRNA remained unchanged (Levallet et al., 2012). This observation raises the possibility that βIII-tubulin translation or turnover may be controlled by K-Ras-induced signalling cascades. In further support of this notion, siRNA knockdown of K-Ras and pharmacologic inhibition of K-Ras downstream effectors resulted in βIII-tubulin protein downregulation (Levallet et al., 2012). Additionally, overexpression of EGFR enhanced βIII-tubulin translation in both K-Ras wild type and mutant expressing cell lines, however non-small cell lung cancer associated EGFR mutations appeared to have no impact on βIII-tubulin translation (Levallet et al., 2012). Understanding, what is driving the increased translation of *TUBB3* in this circumstance would greatly enhance our knowledge on βIII-tubulin translation and stability.

#### 3.2.7 Targeting the *TUBB3* Transcript

Due to the high degree of homology of βIII-tubulin with other β-tubulin isotypes, small molecule inhibitors against this protein are difficult to develop. Given the high expression of *TUBB3*/βIII-tubulin in epithelial cancers, strategies to silence *TUBB3* have been explored. Cui et al. (2020) demonstrated that targeting the *TUBB3* transcript directly was sufficient to restore tumour chemosensitivity. There is strong preclinical evidence that targeting the *TUBB3* transcript through the use of transient or stable gene silencing can increase drug sensitivity, reduce tumour growth, and suppress metastasis in non-small cell lung cancer and pancreatic cancer (Kavallaris et al., 1999; Gan et al., 2007; Gan et al., 2010a; McCarroll et al., 2010; McCarroll et al., 2015b). Along with our colleagues, we have been exploring the development of therapeutic strategies to silence *TUBB3,* and hence βIII-tubulin, in tumors that overexpress this isotype. In pancreatic cancer, we developed polymeric star nanoparticles capable of delivering and potently silencing *TUBB3* siRNA in a clinically relevant orthotopic model of pancreatic cancer and showed that this increased drug sensitivity and reduced metastasis (Teo et al., 2016; McCarroll et al., 2019; Conte et al., 2021). Recently we described the developed of nanoparticles loaded with docetaxel (DTX) and an siRNA against *TUBB3*, in order to have a synergistic effect in the treatment of lung cancer (Conte et al., 2021). In this study we showed that combining DTX/*TUBB3*-siRNA into nanoparticles led to a significant decrease in *TUBB3* and cell viability of tumour cell spheroids compared to nanoparticles loaded with DTX alone—demonstrating the combined anticancer effects of βIII-tubulin reduction and increased drug sensitivity (Conte et al., 2021). Collectively, these studies highlight the potential of developing therapeutic strategies to target *TUBB3* in cancer cells.

## 4 Conclusion

In both normal and cancerous tissue, it is clear the regulation of both *TUBB3* expression and translation is controlled by a complex and multifaced system. This review highlights that a combination of transcriptional controls and altered epigenetic modifications, in conjunction with disrupted signalling pathways may all contribute to disrupted *TUBB3* expression in cancers and subsequent response to therapy. Genomic advances such as single cell analysis and spatial transcriptomics may lead to improved identification of differences between cell-types, and the regulation of *TUBB3* within the tumour microenvironment. Progressing our understanding of βIII-tubulin regulation is not only important in identifying how the nervous system develops but also in cancer, where it will aid in the identification of potential therapeutic targets and treatment strategies.
